# Application of Metabolite-Responsive Biosensors for Plant Natural Products Biosynthesis

**DOI:** 10.3390/bios13060633

**Published:** 2023-06-07

**Authors:** Jianli Zhang, Xinyu Gong, Qi Gan, Yajun Yan

**Affiliations:** School of Chemical, Materials, and Biomedical Engineering, College of Engineering, The University of Georgia, Athens, GA 30602, USA

**Keywords:** plant natural products, biosensor, metabolic engineering

## Abstract

Plant natural products (PNPs) have shown various pharmaceutical activities, possessing great potential in global markets. Microbial cell factories (MCFs) provide an economical and sustainable alternative for the synthesis of valuable PNPs compared with traditional approaches. However, the heterologous synthetic pathways always lack native regulatory systems, bringing extra burden to PNPs production. To overcome the challenges, biosensors have been exploited and engineered as powerful tools for establishing artificial regulatory networks to control enzyme expression in response to environments. Here, we reviewed the recent progress involved in the application of biosensors that are responsive to PNPs and their precursors. Specifically, the key roles these biosensors played in PNP synthesis pathways, including isoprenoids, flavonoids, stilbenoids and alkaloids, were discussed in detail.

## 1. Introduction

In the plant kingdom, higher plants synthesize primary metabolites such as carbohydrate, protein, lipid and organic acid to support basic cell growth and reproduction. Additionally, they produce various secondary metabolites to defend against predators, deliver signals and enhance their tolerance in the environment [1]. A substantial number of them have been identified as plant natural products (PNPs), which exhibit valuable biological activities in human society. PNPs are now widely utilized in the development of pharmaceuticals, cosmetics, food additives and pigments, showing great commercial value in the global market [2]. Traditional methods for PNPs production involve plant extraction and chemical synthesis. However, plant cultivation is labor-intensive, time-consuming, and restricted by soil resources. Chemical synthesis, although enabling fast reactions, presents challenges in synthesizing PNPs with complicated structures in vitro. In addition, the use of toxic reagents, extreme reaction conditions and the generation of hazardous waste still need to be solved [3].

To address these problems, microbial cell factories (MCFs) have been suggested as promising and sustainable approaches for PNPs synthesis. The target pathways can be reconstituted in fast-growing microbial organisms through the introduction of heterogeneous enzymes [4]. However, the synthesis of PNPs follows huge and sophisticated regulation networks in their native hosts, making it challenging for heterogeneous species to grasp the same regulations. Furthermore, the expression of heterogeneous enzymes is sub-optimal and requires codon optimization and gene overexpression, which can disrupt carbon flux balance and cause metabolic burden [5]. To tackle the barriers, scientists have been attempting to mimic the natural regulation systems by employing engineered biosensors into microbes for sustainable bioproduction. Basically, the genetically encoded biosensors are derived from natural regulation elements such as inducible promoters, transcriptional factors (TFs) and riboswitches. Through a series of engineering strategies, the original elements can be adjusted to achieve desired sensitivity and dynamic range, making biosensors powerful tools for strain screening and pathway optimization.

In natural organisms, the majority of genes are subject to intricate regulations. As the most common type of regulatory element, TFs are ubiquitous across various metabolic networks and play a crucial role in coordinating cellular behaviors and maintaining cell homeostasis in response to environmental changes. These TFs are regarded as natural biosensor systems to transduce the environmental signal into transcriptional regulation. Similar to the natural regulation systems, engineered metabolite-responsive biosensors are capable of monitoring the real-time concentration of specific metabolites and converting them into measurable readouts. In general, a typical biosensor system is composed of a regulator protein and a corresponding promoter. The regulator protein contains effector binding sites that can accept potential effectors and DNA binding sites that enable the regulator protein to anchor to the corresponding promoter region. With the binding of effectors, conformational changes happen to the regulator protein, leading to the activation or repression of downstream genes control by corresponding promoters [6]. To optimize the heterologous PNPs biosynthetic pathways in MCFs, metabolite-responsive biosensors are harnessed to dynamically regulate the level and timing of enzyme expression to deal with several metabolic problems [7]. For instance, biosensors can sense certain intermediate and switch on downstream pathways once the intermediate concentration reaches a threshold. This limits downstream pathway expression in the early stages to ensure optimal cell growth, alleviates the pressure caused by the expression of exogenous enzymes, and minimizes the detriments of toxic products. Moreover, with the coupling of CRISPRi and RNAi tools, biosensors can also be applied to repress competing pathways and byproducts formation during the late phase of fermentation, reducing carbon waste on cell growth and other unnecessary routes [8,9]. Therefore, multi-layer dynamic regulations can be established to conquer several metabolic challenges simultaneously by combining multiple biosensor circuits.

**Figure 1:**
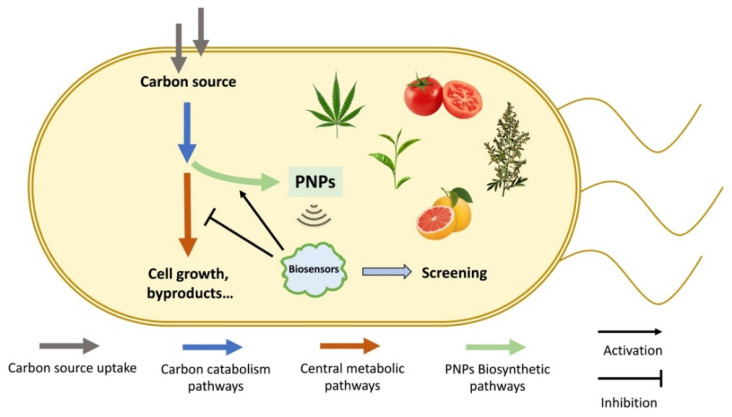
The application of biosensors on plant natural products (PNPs) synthesis in microbial cell factories (MCFs). MCFs have been used to produce various valuable PNPs. Biosensors can be employed to improve production by activating the PNPs synthesis pathways and inhibiting the competition pathways. In addition, these biosensors can also be used for the screening of high-production strains.

Currently, biosensor-based dynamic regulation strategies have been proven to be efficient in optimizing PNPs synthesis in MCFs [10,11]. By using artificial genetic circuits to simulate the natural regulation networks, synthetic pathways can be dynamically adjusted in response to the varying metabolic status, achieving a dynamic balance between cell growth and production and realizing the intelligent carbon flux distribution. In this review, we focused on recent advances in the application of metabolite-responsive biosensors in PNPs synthetic pathways (Figure 1). Especially, the key value-added PNPs types, including isoprenoids, flavonoids, stilbenoids and alkaloids, are discussed in detail.

## 2. Application of Metabolite-Responsive Biosensors on Isoprenoids Production

Isoprenoids are typical PNPs that are composed of multiple isoprene building blocks and play crucial roles in cellular processes such as electron transportation, signaling, photosynthesis and plant defense [12]. In the global market, isoprenoids are widely commercialized as flavorings, pharmaceuticals, fuels and bulk chemicals [13]. Isoprenoids are assembled from the building blocks, which have five carbon atoms, isopentenyl diphosphate (IPP) and its isomer, dimethylallyl diphosphate (DMAPP). Based on the number of carbon atoms, isoprenoids are classified into several types, including C10 monoterpenoids, C20 diterpenoids, C25 sesterterpenoids, C30 triterpenoids, C40 tetraterpenoids and even polyterpenoids with more than forty carbon atoms [14]. Due to the high value of isoprenoids, efforts have been made to develop isoprenoid synthetic pathways in MCFs. There are two routes to synthesize IPP and DMAPP: the mevalonate (MVA) pathway, which mainly occurs in eukaryotes such as yeast, and the methylerythritol phosphate (MEP) pathway, which mainly exists in prokaryotes such as *E. coli*. Both pathways have been employed in engineered MCFs for isoprenoid bioproduction. However, the MEP pathway usually requires optimization to achieve high yield due to an insufficient supply of IPP and DMAPP [15]. Subsequently, the integration of small units results in longer chain intermediates such as C10 geranyl diphosphate (GPP), C15 farnesyl diphosphate (FPP), and C20 geranylgeranyl diphosphate (GGPP), which are all important precursors for the further synthesis of various isoprenoids. Nevertheless, some heterologous enzymes introduced into engineered microbes may have suboptimal performance within the long synthesis routes. In addition, producing complicated isoprenoids often leads to issues such as metabolic stress, growth retardation, and low yield due to the imbalance of carbon flux [16]. Biosensor systems have emerged as powerful tools for addressing these problems in isoprenoid production. As the engineered biosensors can detect specific intermediates or the final products, researchers were motivated to develop different biosensors for high producer screening or dynamic regulation.

**Figure 2:**
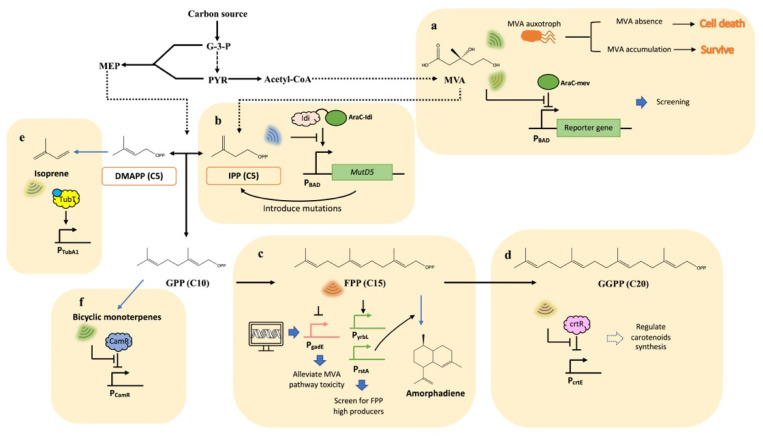
The biosynthesis pathways of isoprenoids and the biosensors that are responsive to isoprenoids and their precursors. (**a**) The mevalonate (MVA)-responsive biosensors. The MVA auxotroph strain survived only when the MVA was supplied. Otherwise, the absence of isopentenyl diphosphate (IPP) and dimethylallyl diphosphate (DMAPP) will cause growth defects. The AraC variant AraC-mev was able to inhibit the corresponding promoter and can be depressed by MVA. (**b**) The IPP-responsive biosensor. The engineered AraC was coupled with Idi enzyme and can activate the transcription of the corresponding promoter. The activation process was inhibited by IPP. The downstream *MutD5* is a mutator; when the mutations which enhance IPP supply were generated, the expression of the mutator will be inhibited. (**c**) The farnesyl diphosphate (FPP)-responsive biosensors. P_gadE_ was identified to be inhibited by FPP, while P_yrbL_ and P_rstA_ were identified to be activated by FPP. These biosensors have been applied to regulate the MVA pathways and screen for high producers. (**d**) The geranylgeranyl diphosphate (GGPP)-responsive biosensor. crtR is a repressor to the corresponding promoter and can be released with the binding of GGPP. This biosensor has the potential to regulate downstream carotenoid biosynthesis. (**e**) The Isoprene-responsive biosensor. TubT activates the transcription of the corresponding promoter with the binding of isoprene. (**f**) Bicyclic monoterpenes-responsive biosensor. The transcriptional repressor CamR was engineered to respond to some bicyclic monoterpenes. G-3-P, glycerol-3-phosphate; PYR, pyruvate.

The MVA pathway initiates from the central metabolite acetyl-CoA. Owing to the high availability of precursors, the MVA pathway is usually introduced into the non-MVA pathway hosts for downstream isoprenoid production [17]. As the key intermediate of the MVA pathway, mevalonate can serve as an indicator of both the expression level of the MVA pathway and the capacity of terpenoid production. The development of mevalonate biosensors has been ongoing for several decades. Although TF-based biosensors that respond to mevalonate have not been identified, alternative methods were employed to detect mevalonate accumulation. For instance, Pfleger et al. constructed a mevalonate biosensor strain by creating an *E. coli* mevalonate auxotroph (Figure 2a). The heterologous MVA pathway was partially introduced into *E. coli* to enable the cell to convert exogenous mevalonate to IPP and DMAPP, which are necessary for cell survival. Then, the endogenous source of IPP and DMAPP was cut off by disrupting the native MEP pathway. A reporter plasmid including constitutively expressed *egfp* was transformed, and the engineered biosensor strain was able to survive once exogenous mevalonate was supplied. Thus, the fluorescence level indicated the mevalonate concentration [18]. In addition to constructing auxotrophic strain, modifying the substrate specificity of known transcriptional factors is another approach to engineering mevalonate biosensors. The L-arabinose-responsive homodimeric protein AraC is a repressor to promoter P_BAD_. Tang et al. acquired a mevalonate-responsive AraC protein variant through site saturation mutagenesis (Figure 2a). The AraC-mev variant was able to activate the transcription at P_BAD_ with the binding of mevalonate, and the response to mevalonate was linear in the range of 10–100 mM. In this study, AraC-mev was employed to screen for mutants with optimally expressed HMG-CoA reductase (tHMGR), which is responsible for mevalonate synthesis [19]. In another study, the AraC-mev expression cassette was fine-tuned to work in the methylotrophic model strain *M. extorquens* AM1, serving for the high-throughput screening of the regulator QscR. The engineered biosensor had a linear relationship in the range of 0–1.7 mM mevalonate. By using this screening platform, a mutant strain Q49 with a mevalonate yield of up to 2.67 g/L was obtained [20].

IPP is the end product of the MVA and MEP pathways, serving as the C5 starting unit for over 50,000 isoprenoids. Monitoring cellular IPP concentration can provide insight into the capacity of the isoprenoids building blocks pool. Isopentenyl pyrophosphate isomerase (Idi) has been shown to adopt different conformational statuses when binding with IPP. Based on this feature, Chou et al. designed an IPP biosensor by fusing the AraC DNA binding domain with Idi via a short peptide linker (Figure 2b). This biosensor prevented the activation of the P_BAD_ promoter in the presence of IPP due to a conformational change in the synthetic transcriptional factor. The IPP biosensor was then applied to a feedback-regulated evolution of phenotype (FREP) platform to increase lycopene production. In this study, the mutator-encoded gene *MutD5* was placed under the regulation of P_BAD_, which can generate mutations on the chromosome in the absence of the ligand. Once mutations that facilitate IPP accumulation occur, P_BAD_ would be inhibited to prevent further evolution, and the expected mutations that contribute to higher lycopene production were conserved in the strain [21].

The C15 intermediate FPP is a precursor for the synthesis of many value-added isoprenoids such as farnesene, amorphadiene and squalene. IPP and FPP can be toxic when accumulating in *E. coli*, leading to impaired cell growth and reduced productivity [22]. In previous research, Dahl et al. characterized several promoters that are sensitive to the toxicity of FPP through whole-genome transcriptional analysis (Figure 2c). Specifically, P_rstA_ and P_yrbL_ were activated by FPP, while P_gadE_ was repressed. Using the P_gadE_-regulated MVA pathway, the titer of an artemisinin precursor, amorphadiene, was increased two-fold [23]. In order to improve the biosynthesis of zeaxanthin, a food and feed additive, Shen et al. employed the previously reported FPP biosensor P_rstA_ to screen for high FPP producers from tunable intergenic regions (TIGRs) libraries which was integrated into the MVA pathway. P_gadE_ was also used to dynamically control MVA pathway genes to efficiently alleviate the toxicity caused by FPP accumulation, and the fed-batch fermentation yielded up to 722.46 mg/L of zeaxanthin [24].

The enzyme farnesyltranstransferase catalyzes the synthesis of C20 isoprenoid GGPP, which is the precursor for various carotenoids such as lycopene, astaxanthin and zeaxanthin. However, the cellular GGPP concentration is relatively low, necessitating biosensors with high sensitivity for detection. The MarR-type transcriptional regulator, crtR, represses the carotenogenic gene cluster and could be derepressed in the presence of GGPP [25]. Henke et al. characterized crtR from *Corynebacterium glutamicum* as a GGPP biosensor (Figure 2d). This biosensor was able to effectively monitor GGPP accumulation between 0.1 and 4 mM, providing a potentially valuable tool for optimizing carotenoid production [26].

In addition to the main intermediates on the isoprenoid pathway, ergosterol can be utilized as an indicator outside of the isoprenoid pathway for enhancing production. Ergosterol is an essential compound for the basic functions of fungal cells, and both ergosterol and its precursor squalene are derived from FPP, which competes for FPP supply with the heterologous isoprenoid synthetic pathways. Amiri et al. substituted the native promoter of the squalene-synthase-encoded gene, *Erg9*, with the methionine-dependent repressible *MET3* promoter. By preventing the release of byproduct squalene, the production of linalool was improved to 95 μg/L when methionine was added [27]. Yuan et al. identified four ergosterol-responsive repression promoters according to the result of qRT-PCR. When the native *Erg9* promoter was replaced with an ergosterol-responsive promoter P_Erg1_, the production of amorpha-4,11-diene showed up to a fivefold improvement compared to the control [28]. Similarly, Callari et al. replaced the promoters of *Erg9* with P_Erg1_, resulting in an increased casbene titer [29]. In another study, Ignea et al. used P_Erg1_ to regulate the farnesyl-diphosphate-synthase-encoded gene *Erg20* for dynamically driving more IPP and DMAPP to the engineered monoterpenoids biosynthesis pathways [30].

In recent years, there has been a growing interest in the design of genetically encoded biosensors that respond to a variety of simple isoprenoids. Kim et al. developed an isoprene biosensor based on the XylR-type transcriptional factor TbuT from *Ralstonia pickettii* (Figure 2e). The engineered biosensor showed a linear relationship in the range of 0.05 mM to 8 mM isoprene. In the presence of isoprene, TubT was activated, turning on the expression of P_TubA1_ both in *E. coli* and *P. putida* [31]. d’Oelsnitz et al. designed a biosensor for bicyclic monoterpenes using the camphor-responsive transcriptional factor CamR from *P. putida* (Figure 2f). CamR is a TetR-family regulator. After several rounds of directed evolution, they modified the CamR variants to create a generalist bicyclic monoterpenes biosensor system that responds to borneol, fenchol, eucalyptol and camphene [32].

## 3. Development and Application of Flavonoids-Responsive Biosensors

**Figure 3:**
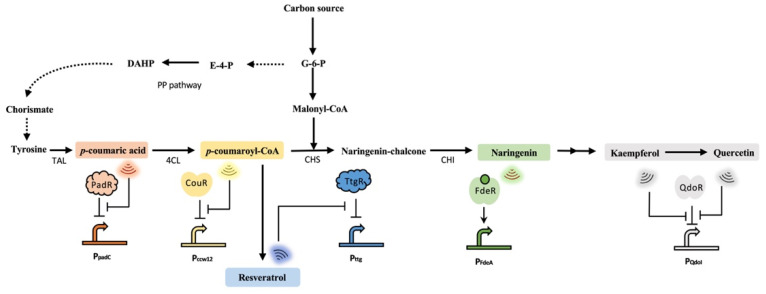
The biosynthesis pathways of typical flavonoids and stilbenoids and the related biosensors. The biosynthesis of flavonoids and stilbenoids is derived from the shikimate pathway. Many biosensors have been developed to sense the key intermediates, including the *p*-coumaric acid-responsive biosensor PadR, the *p*-coumaroyl-CoA-responsive biosensor CouR and the naringenin-responsive biosensor FdeR. Flavonoid-responsive biosensors are also designed. TtgR was engineered to sense resveratrol, FdeR was designed to sense naringenin and QdoR was engineered to sense kaempferol and quercetin. G-6-P, glucose-6-phosphate; E-4-P, erythrose 4-phosphate; DAHP, 3-deoxy-arabino-heptulosonate 7-phosphate; PP pathway, pentose phosphate pathway.

Flavonoids are important polyphenolic PNPs that are widely distributed in vascular plants, and they are also responsible for flavor, color, and pharmacological activities [33]. Flavonoids are structurally derived from *p*-coumaric acid or ferulic acid and consist of a 15-carbon (C6-C3-C6) backbone. With over 9000 plant flavonoids identified so far, they have gained much attention for their potential biological properties, such as anti-oxidative, anti-inflammatory, anti-cancer, antimicrobial, anti-carcinogenic and vascular activities [34,35]. Currently, the estimated global market for flavonoids can reach USD 200 million annually [36]. However, conventional plant extraction methods failed to meet the increasing market demand due to their limitations, such as the long lifecycle of plants. Comparatively, metabolic engineering provides a sustainable and efficient approach in terms of producing flavonoids [36]. Recently, engineered microorganisms such as *Escherichia coli*, *Corynebacterium glutamicum*, *Saccharomyces cerevisiae*, *Yarrowia lipolytica*, and *Lactococcus lactis* have been reported to achieve heterogeneous production of flavonoids by introducing or reconstructing related biosynthetic pathways [37,38,39]. In MCFs, flavonoids are synthesized from tyrosine via the shikimate pathway. Tyrosine ammonia lyase (TAL) and 4-coumarate CoA ligase (4CL) are two critical enzymes responsible for converting tyrosine to *p*-coumaroyl-CoA. By adding three malonyl-CoA moieties, *p*-coumaroyl-CoA is then converted to naringenin chalcone catalyzed by chalcone synthase (CHS). The cyclization of naringenin chalcone by chalcone isomerase (CHI) yields naringenin, which serves as the gateway compound for the synthesis of other complex flavonoids (Figure 3).

Despite the potential of metabolic engineering, several critical bottlenecks need to be addressed, including identifying production strains, balancing metabolism, managing competition between production and cell growth, and handling the accumulation of toxic intermediates and byproducts. Researchers have used biosensor systems to detect and quantify the production of flavonoids or intermediates in real time, which has allowed for the rapid screening of high-producing strains [40]. For example, the PadR-P_padC_ biosensor system is a common tool that has been applied in high-throughput screening for high-producing strains of *p*-coumaric acid or ferulic acid (Figure 3). The dynamic range of the PadR-P_padC_ biosensor system can be adjusted through RBS engineering and protein engineering strategies. Siedler et al. conducted a dynamic range test on the PadR-P_padC_ biosensor system by altering its ribosomal binding site. Subsequently, they successfully encapsulated the yeast producers with the *E. coli* cells that harbored biosensor systems to rapidly screen *p*-coumaric acid high-producing variants [41]. Jiang et al. optimized the PadR-P_padC_ biosensor system for versatile dynamic performance via site-directed PadR evolution and the construction of hybrid promoters. In contrast to wild-type PadR (0–600 mg/L), mutant K64A displayed a broader operating scope (0–1000 mg/L), and mutant H38A is very sensitive and can be activated by as little as 5 mg/L *p*-coumaric acid [42]. The optimized PadR(K64A)-P_padC_ biosensor system was further applied in dynamic regulation and was found to be effective in enhancing the supply of *p*-coumaric acid [43].

Naringenin belongs to the flavanones subclass. Key intermediates such as *p*-coumaroyl-CoA and malonyl-CoA are critical for the synthesis of naringenin. The LysR-type transcriptional activator FdeR from *Herbaspirillum seropedicae* was demonstrated to respond to naringenin (Figure 3) [44]. Wang et al. optimized FdeR to create a biosensor system that exhibited outstanding performance in identifying *S. cerevisiae* strains with high naringenin production [45]. Achieving optimal flavonoid production requires a careful balance of multiple factors. Genetically encoded biosensors have been developed and applied for dynamic regulation networks, enabling the autonomous reallocation of carbon fluxes in response to the key intermediates to facilitate the production of target compounds. By combining the PadR system and the FdeR system, Zhou et al. operated multilevel dynamic regulation on the malonyl-CoA metabolic pathway and achieved an 8.7-fold increase in (2*S*)-naringenin production [46]. In another study, Jiang et al. established an autonomous cascaded artificial dynamic (AutoCAD) regulation circuit to keep pathways balanced based on the PadR and FdeR biosensor systems, increasing naringenin titer by 16.5-fold. In fed-batch fermentation, the naringenin titer can reach 277.2 mg/L [11]. As a central precursor for many flavonoids, *p*-coumaroyl-CoA was regarded as an ideal effector for dynamic regulation. Liu et al. developed a novel biosensor CouR in *Saccharomyces cerevisiae* that responds to *p*-coumaroyl-CoA (Figure 3). CouR is a MarR-type transcriptional repressor in bacteria. As *p*-coumaroyl-CoA could not be detected using standard analytical methods, the concentration threshold and operational range of the sensor were not able to be quantified. Nevertheless, a dynamic regulatory circuit was developed by Liu et al. to adjust the production of *p*-coumaroyl-CoA in real time. By combining CouR with the malonyl-CoA biosensor FapR, the dual-regulation of *p*-coumaroyl-CoA synthesis within the naringenin biosynthesis pathway was achieved. The naringenin titer reached 47.3 mg/L upon external precursor feeding, displaying a 15-fold increase relative to the non-regulated system [47].

The different categories of flavonoids are formed through the further modification of flavanones. Flavanonol is a critical intermediate and a key branch point in the flavonoid biosynthesis pathway. Dihydroflavonols are common precursors for flavonol biosynthesis and are produced from flavanones catalyzed by the enzyme flavanone 3-hydroxylase (F3H). F3H catalyzes the conversion of naringenin, sageol and pentahydroxyflavanones to the corresponding dihydromyricetin (DHK), dihydroquercetin (DHQ) and dihydromyricetin (DHM) products. The production titers, rates, and yields of pterostilbene, kaempferol, and quercetin still need to be improved for industrial applications. Siedler et al. exploited the QdoR biosensor system from *Bacillus subtilis*. As a TetR-family transcriptional repressor, QdoR can be derepressed by at least 0.01 mM quercetin or at least 0.005 mM kaempferol (Figure 3). This biosensor system was successfully applied to detect kaempferol production in vivo. Additionally, the QdoR-derived biosensor was expected to help identify genes involved in flavonoid biosynthesis, also enable the real-time detection of kaempferol levels and enhance its production [48].

## 4. Application of Stilbenoids-Responsive Biosensors

Stilbenoids, also known as stilbenes, are produced through the catalysis of *p*-coumaroyl-CoA and malonyl-CoA by stilbene synthase (STS). This process represents the initial branch of the flavonoid biosynthetic pathway. In nature, plants use stilbenes to defend against microbial infections. Resveratrol, a type of stilbene, has gained significant commercial interest due to its broad range of biological activities. Extensive efforts have been dedicated to producing resveratrol using metabolic engineering techniques in microbial hosts such as *Escherichia coli* and *Saccharomyces cerevisiae*. TtgR is a TetR-type transcriptional repressor that responds to antibiotics, flavonoids and organic solvents [40,49,50]. Xiong et al. modified TtgR to respond to resveratrol (Figure 3). Furthermore, the engineered TtgR was used to screen for 4CL variants with enhanced activity from a 4CL mutagenesis library, and the resulting mutants were demonstrated to increase the production of resveratrol and naringenin. The findings indicated that it is possible to enhance the activity of crucial enzymes in significant biosynthetic pathways by using custom-designed biosensors [51]. According to the genome mining of the *Novosphingobium aromaticivorans* DSM 12444, Sun et al. discovered a new MarR family stilbene-responsive biosensor Saro_0803. The naturally occurring TF-promoter pair was able to be introduced into *E. coli* and can respond to various compounds, such as resveratrol. The biosensor demonstrated efficient monitoring of resveratrol accumulation within the range of 0.2–0.8 mM. It can even tell resveratrol apart from other similar compounds. This type of biosensor also has the potential to aid in the development of highly efficient screening processes for strains that produce stilbenes or even cannabinoids [52].

## 5. Application of Metabolite-Responsive Biosensors on Alkaloids Production

**Figure 4:**
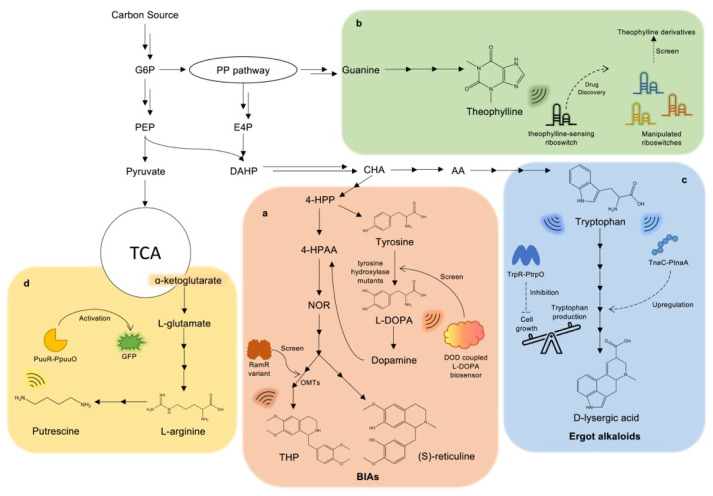
The biosynthesis pathways of various alkaloids and their corresponding responsive biosensors. (**a**) The biosynthesis of benzylisoquinoline alkaloids (BIAs) is shown along with the applications of DOD-coupled L-DOPA biosensor and RamR variant to enhance the production of (*S*)-reticuline and THP, respectively. Specifically, the DOD-coupled L-DOPA biosensor was used to screen tyrosine hydroxylase mutants in (*S*)-reticuline synthesis pathway, while RamR, a THP responsive biosensor, was utilized to screen O-methyltransferases (OMTs) in THP synthesis pathway. (**b**) The biosynthesis of theophylline and the potential application of a theophylline-sensing riboswitch. Riboswitches are easily manipulable, and therefore, manipulated theophylline-sensing riboswitches hold the potential to be applied in drug discovery of theophylline derivatives. (**c**) The biosynthesis of tryptophan-derived ergot alkaloids and the possible utilization of tryptophan-responsive biosensors. TrpR, a transcriptional repressor, can be used to balance cell growth and tryptophan production to provide sufficient precursor. TnaC, a leading peptide, acts as an activator to upregulate the synthases of D-lysergic acid. (**d**) The biosynthesis of putrescine is presented along with the application of a putrescine-responsive biosensor. PuuR is capable of detecting the titer of putrescine in real-time by testing PpuuO-controlled GFP readout. Abbreviation: G6P, glucose-6-phosphate; PEP, phosphoenolpyruvate; E4P, D-erythrose 4-phosphate; DAHP, 3-deoxy-D-arabino-heptulosonate-7-phosphate; CHA, Chorismate; AA, Anthranilate; 4HPP, 4-hydroxyphenylpyruvate; 4-HPAA, 4-hydroxyphenylacetaldehyde; NOR, norlaudanosoline; THP, tetrahydropapaverine. BIAs, benzylisoquinoline alkaloids; PP pathway, Pentose phosphate pathway. One solid arrow, one-step reaction; Two solid arrows, multiple-step reactions.

Alkaloids are a class of organic nitrogen-containing natural products existing in terrestrial and marine organisms. The wide distribution of alkaloids leads to the formation of various chemical structures, from simple-chain to complicated multiple-ring structures. These structures are mainly derived from the L-amino acid of living beings, including L-arginine, L-tyrosine, L-tryptophan, etc. [53]. This class of natural products is a hot spot in drug discovery and development due to the high and diverse biological activities associated with anti-bacterial, anti-inflammatory, and anti-cancer properties, which have the potential to overcome multiple drug resistance (MDR) and treat rare diseases [54]. The conventional manufacturing of alkaloids relies on the extraction from natural resources, but the long-life cycle of plants has hindered production [55]. As a result, current manufacturing cannot meet the demands of the pharmaceutical markets. With the development of metabolic engineering and synthetic biology, microbes have become promising hosts for alkaloid bioproduction. In 2022, the FDA-proved anti-cancer drug vincristine, a type of monoterpene indole alkaloids (MIAs), was successfully synthesized using engineered yeast. The de novo biosynthesis titer of vincristine precursors, catharanthine and vindoline, reached 91.4 μg/L and 13.2 μg/L, respectively, which was a milestone in the biocatalysis of MIAs and shed light on the biosynthesis of large complex natural products via MCFs [56]. In the face of drug shortages, programable microbes, such as *E. coli* and yeast, can help achieve high-yield drug production and high-throughput drug screening by adopting heterogeneous enzymes and applying synthetic biology tools. The engineered metabolite-responsive biosensors act as actuators or indicators in the metabolism regulation of microorganisms (Figure 4). Moreover, studies have indicated that synthetic biosensors enable high-yield drug production by dynamically regulating precursors or drug synthetic pathways, as well as making high-throughput drug screening visible by triggering fluorescence proteins.

Plant alkaloids are large families of valuable drugs, and benzylisoquinoline alkaloids (BIAs) are a particular type with complex condensed ring structures that are derived from L-tyrosine or L-DOPA [57]. The paradigm products included morphine, papaverine, and noscapine [58]. In recent years, the key enzymes for BIAs biosynthesis have been explored in plants and employed in *E. coli* or yeast for high-yield bioproduction. To improve the production of BIAs, DeLoache et al. reported an enzyme-coupled biosensor in yeast to screen a yeast-active tyrosine hydroxylase, the step-limiting enzyme for (*S*)-reticuline biosynthesis. The designed biosensor coupled a plant-originated DOPA dioxygenase (DOD) that can catalyze L-DOPA into yellow-colored betaxanthin in the operational range of 2.5 μM to 2500 μM L-DOPA, and then the yellow fluorescence of the pigment was utilized as the indicator for tyrosine hydroxylases screening (Figure 4a). Meanwhile, to reduce the generation of byproducts, the authors further constructed a violet pigment betanidin indicator for second-round tyrosine hydroxylase mutant screening to find a variant with reduced DOPA oxidase activity. The selected tyrosine hydroxylase mutant (CYP76AD1^W13L F309L^) showed 2.8-fold greater L-DOPA production and significantly decreased DOPA oxidase activity compared to the wild-type. After combining production modules with the optimized enzyme, the production of (*S*)-reticuline from glucose reached 80.6 μg/L in 96 h fermentation in shake flasks [59]. Except for enzyme-coupled biosensors, transcriptional factor-based biosensors for BIAs were also characterized recently. RamR is a TetR-type multidrug-resistance regulator found in *Salmonella* typhimurium [60]. It is promising to engineer the multidrug-resistance regulator to a BIAs-sensing biosensor since the regulator has the potential to respond to several natural alkaloids. Based on this hypothesis, d’Oelsnitz and colleagues developed a series of fungible biosensors for alkaloid sensing (Figure 4a). The initial fluorescence test of five BIAs (tetrahydropapaverine (THP), papaverine (PAP), rotundine (ROTU), glaucine (GLAU) and noscapine (NOS)) to RamR showed only modest activation. To improve the affinity between BIAs and RamR, five semi-rational libraries were screened by RamR-based seamless enrichment of ligand-inducible sensors (SELIS). RamR variants with over 100-fold specificities and 100 μM operational ranges for their cognate ligands were then selected through the use of SELIS. The crystal structures further revealed the detailed interactions between BIAs and regulators, explaining the outstanding responsive performance of RamR mutants. Finally, the evolved BIAs biosensors were used to screen O-methyltransferases (OMTs) mutagenesis libraries for improved catalytic activity to achieve tetrahydropapaverine (THP) biosynthesis. Additionally, three selected OMT variants (GEN3, GEN4 and GEN5) with engineered substrate-binding pockets were able to produce THP with the titers of 0.21 mg/L, 1.48 mg/L and 0.89 mg/L, respectively [10].

Cannabinoids (CBD) are also a category of plant-derived alkaloids with complex chemical structures. They play a vital role in several physiological processes of the human body through the endocannabinoid system and have proven to be efficient in treating neurological diseases [61]. The biosynthesis of CBD in plants was clearly identified and classified into enzyme modules, which provided hints for the bioproduction in microbes [62]. By introducing *Cannabis* gene modules and other functional optimized homologous genes into yeast, Luo et al. successfully constructed the biosynthesis pathway from simple sugar galactose to CBD and its analogues. The engineered yCAN53 strain was able to produce 8.0 mg/L tetrahydrocannabinolic acid (THCA) and 4.8 mg/L tetrahydrocannabivarinic acid (THCVA), respectively, paving the way for high-yield CBD bioproduction [63]. Biosensor-coupled dynamic regulation is an attractive approach to achieving high-yield production. A newly characterized G-protein-coupled receptors (GPCR)-based yeast biosensor with the ability to detect CBD was developed, connecting the yeast pheromone-signaling pathway to human canonical cannabinoid receptor (CB2) to form a yeast whole-cell biosensor. The biosensor exhibited high affinities and maximum signal-to-noise ratio (SNR) to CBD and its analogues. Finally, the authors developed a portable CBD biosensor-based detector that was sensitive to the presence of cannabinoids in real-life samples. For example, the detectable range of the canonical cannabinoid THC was 30 pM–100 µM. Therefore, it was also possible to utilize this biosensor for the dynamic regulation of CBD production in yeast [64]. Except for the complicated plant-derived alkaloids, there are some alkaloids with simple chemical structures. Theophylline and its isomers are originated from cocoa and green tea and rich in beverages [65]. The immunomodulatory and cardioprotective effects made this compound valuable for medical use. Wachsmuth et al. devised a theophylline-sensing riboswitch through computational approaches (RNAfold) and characterized it both in vitro and in vivo (Figure 4b). The binding of theophylline to the aptamer formed a stable stem structure and destroyed the terminator in front of the transcriptional start site of the gene, resulting in an ON stage of transcription. The optimized RS10shift riboswitch exhibited a 6.5-fold ON/OFF rate in response to 2 mM theophylline. Due to the flexible manipulation of riboswitch, the theophylline-sensing riboswitch could be applied to drug screening for theophylline derivatives [66].

Ergot alkaloids are another family of alkaloids that originate from fungi. In medical treatments, ergot alkaloids have shown positive effects on neutral diseases, such as migraines and Parkinson’s disease, which has led to increased demands for their large-scale production [67]. To fulfill the efficient bioproduction, Wong et al. reported a biosynthesis method for ergoline derivatives in yeast. The engineered DLAM33B strain was able to produce 1.7 mg/L and 1.4 mg/L D-lysergic acid (DLA) in the 1L- and 4L-fermentations, respectively [68]. According to the DLA biosynthesis pathway, L-tryptophan is an important building block of ergot alkaloids. For this reason, coupling L-tryptophan-responsive biosensors with ergot alkaloids biosynthesis pathway would be beneficial to actuate high-yield production (Figure 4c). TrpR is a typical transcriptional factor of *E. coli* and is inherently in charge of aromatic biosynthesis pathways. L-tryptophan is the effector of TrpR and triggers the repression of the *trpO* operator [69]. For instance, the engineered TrpR1(V58E) variants showed a preferred response to tryptophan in the operational range from 0 to 30 mg/L; and the engineered TrpR1(V58K) was favorable to sense 5-hydroxytryptophan in the operational range from 0 to 125 mg/L [70]. Although the mechanisms and structures of TrpR-based biosensors were well-characterized with diverse dynamic performance and ligand-specificity, they have rarely been applied to metabolic pathways as the single repression strategy was limited for the pathway regulation design. However, TrpR repressor still has the potential to help reduce byproducts and balance cell growth and aromatics production to increase carbon flux towards ergot alkaloids biosynthesis. Alternatively, TnaC, a leading peptide with activation mechanisms in *E. coli*, could be used instead of the TrpR repressor. In the presence of L-tryptophan, TnaC was able to interact with it, leading to the transcriptional activation of downstream genes. The TnaC variant (R23H) was able to trigger the maximum signal output at around 0.1 mM tryptophan [71]. Thus, TnaC-based biosensors could be used to boost the downstream ergot alkaloid synthesis pathway when L-tryptophan accumulates. Although further engineering work is needed to apply these biosensors to real-life fermentation, it is feasible to use L-tryptophan-sensing biosensors to aid in the production of ergot alkaloids.

Putrescine is a polyamine alkaloid that serves as a raw material for bulk chemicals, such as nylon 46 [72]. Moreover, putrescine is an important precursor of tropane alkaloids in biosynthesis. To monitor putrescine production in *E. coli*, Chen et al. borrowed the native putrescine-responsive PuuR repressor for biosensor engineering (Figure 4d). The inducing of putrescine relieved the repression of PuuR on its responsive promoter, leading to a dose-dependent increase in green fluorescence protein (GFP) output within the range of a wide range of 0.048–18.049 mg/g DW. After implementing the biosensor into the host strain, the relation between putrescine titer and GFP was investigated and plotted, reflecting the increased fluorescence density as putrescine concentration increased. Therefore, this biosensor could quantitatively describe changes in putrescine levels in a dynamic manner by GFP, making it possible to monitor MCFs in real time. Additionally, the tunable PuuR-based putrescine-sensing biosensor has the potential to serve as an essential component for high-throughput screening or a dynamic regulation platform for polyamine alkaloids and tropane alkaloids biosynthesis [73].

## 6. Conclusions and Perspectives

In recent decades, significant advancements have been made in establishing synthetic pathways for a variety of PNPs in MCFs, accompanied by the application of versatile genetically encoded biosensors. This paper provided a comprehensive overview of the progress in the development and application of biosensors that are responsive to PNPs and their related metabolites for high producer screening and dynamic regulation (Table 1). Since most PNPs with valuable bioactivities possess complicated chemical structures, their biosynthesis typically requires the expression of multiple exogenous enzymes, as well as a large amount of energy and cellular resources to fulfill long pathways, which can impose much metabolic burden on the host cell. Until now, biosensor-aided metabolic engineering strategies have made remarkable contributions to optimizing the biosynthesis of PNPs, including but not limited to isoprenoids, flavonoids, stilbenoids and alkaloids.

Despite the progress in the investigation of biosensors for PNPs recognition, the number of available biosensors is still limited compared to the vast number of PNPs species. To enrich the biosensor toolbox, protein engineering paves the way for expanding substrate specificity and fine-tuning the profile of existing biosensors. With the rapid development of advanced computational techniques, such as molecular dynamics (MD) simulation and machine learning prediction models, such as Alpha-fold [74,75], the process of biosensor engineering can be significantly accelerated. In addition, omics and genome mining tools can facilitate the identification of new potential biosensors. In the future, we expect that the discovery and optimization of PNP-responsive biosensors will be standardized and high-speed with the assistance of computational methods.

## Figures and Tables

**Table 1:** Biosensors with the potential to enhance or that have been utilized to improve the production of plant natural products (PNPs).

PNPs	Biosensors	Mechanisms	Effectors	References
Isoprenoids	*E. coli* mevalonate auxotroph	Whole cell biosensor	Mevalonate	[18]
	AraC-mev	TFs	Mevalonate	[19,20]
	IA	TFs	IPP	[21]
	P_rstA_	Responsive promoter	FPP	[23,24]
	P_yrbL_	Responsive promoter	FPP	[23]
	P_gadE_	Responsive promoter	FPP	[23,24]
	crtR	TFs	GGPP	[25,26]
	P_Erg1_	Responsive promoter	Ergosterol	[28,29,30]
	TubT	TFs	Isoprene	[31]
	CamR	TFs	Bicyclic monoterpenes	[32]
Flavonoids	PadR	TFs	*p*-coumaric acid	[11,41,42,43,46]
	FdeR	TFs	Naringenin	[11,44,45,46]
	CouR	TFs	*p*-coumaroyl-CoA	[47]
	QdoR	TFs	Quercetin and kaempferol	[48]
Stilbenoids	TtgR	TFs	Resveratrol	[51]
	Saro_0803	TFs	Resveratrol	[52]
Alkaloids	DOD	Enzyme-coupled biosensor	L-DOPA	[59]
	RamR	TFs	BIAs	[10]
	CB2	GPCR	CBD	[64]
	RS10shift	Riboswitch	Theophylline	[66]
	TrpR	TFs	L-tryptophan	[69,70]
	TnaC	Leader peptide	L-tryptophan	[71]
	PuuR	TFs	Putrescine	[73]

## Data Availability

No new data were created or analyzed in this study. Data sharing is not applicable to this article.

## References

[1] Kroymann J. (2011). Natural diversity and adaptation in plant secondary metabolism. Curr. Opin. Plant Biol..

[2] Wang H., Guo H., Wang N., Huo Y.X. (2021). Toward the Heterologous Biosynthesis of Plant Natural Products: Gene Discovery and Characterization. ACS Synth. Biol..

[3] Atanasov A.G., Waltenberger B., Pferschy-Wenzig E.M., Linder T., Wawrosch C., Uhrin P., Temml V., Wang L., Schwaiger S., Heiss E.H. (2015). Discovery and resupply of pharmacologically active plant-derived natural products: A review. Biotechnol. Adv..

[4] Cravens A., Payne J., Smolke C.D. (2019). Synthetic biology strategies for microbial biosynthesis of plant natural products. Nat. Commun..

[5] Li C., Jiang T., Li M., Zou Y., Yan Y. (2022). Fine-tuning gene expression for improved biosynthesis of natural products: From transcriptional to post-translational regulation. Biotechnol. Adv..

[6] Maddocks S.E., Oyston P.C.F. (2008). Structure and function of the LysR-type transcriptional regulator (LTTR) family proteins. Microbiology.

[7] Teng Y., Zhang J., Jiang T., Zou Y., Gong X., Yan Y. (2022). Biosensor-enabled pathway optimization in metabolic engineering. Curr. Opin. Biotechnol..

[8] Yang Y., Lin Y., Wang J., Wu Y., Zhang R., Cheng M., Shen X., Wang J., Chen Z., Li C. (2018). Sensor-regulator and RNAi based bifunctional dynamic control network for engineered microbial synthesis. Nat. Commun..

[9] Wang J., Zhang R., Zhang J., Gong X., Jiang T., Sun X., Shen X., Wang J., Yuan Q., Yan Y. (2021). Tunable hybrid carbon metabolism coordination for the carbon-efficient biosynthesis of 1,3-butanediol inEscherichia coli. Green Chem..

[10] d’Oelsnitz S., Kim W., Burkholder N.T., Javanmardi K., Thyer R., Zhang Y., Alper H.S., Ellington A.D. (2022). Using fungible biosensors to evolve improved alkaloid biosyntheses. Nat. Chem. Biol..

[11] Jiang T., Li C., Zou Y., Zhang J., Gan Q., Yan Y. (2022). Establishing an Autonomous Cascaded Artificial Dynamic (AutoCAD) regulation system for improved pathway performance. Metab. Eng..

[12] Lange B.M., Rujan T., Martin W., Croteau R. (2000). Isoprenoid biosynthesis: The evolution of two ancient and distinct pathways across genomes. Proc. Natl. Acad. Sci. USA.

[13] Tetali S.D. (2019). Terpenes and isoprenoids: A wealth of compounds for global use. Planta.

[14] Kirby J., Keasling J.D. (2009). Biosynthesis of plant isoprenoids: Perspectives for microbial engineering. Annu. Rev. Plant Biol..

[15] Du F., Yu H., Xu J., Li C. (2014). Enhanced limonene production by optimizing the expression of limonene biosynthesis and MEP pathway genes in E. coli. Bioresour. Bioprocess.

[16] Vickers C.E., Williams T.C., Peng B., Cherry J. (2017). Recent advances in synthetic biology for engineering isoprenoid production in yeast. Curr. Opin. Chem. Biol..

[17] Morrone D., Lowry L., Determan M.K., Hershey D.M., Xu M., Peters R.J. (2010). Increasing diterpene yield with a modular metabolic engineering system in E. coli: Comparison of MEV and MEP isoprenoid precursor pathway engineering. Appl. Microbiol. Biotechnol.

[18] Pfleger B.F., Pitera D.J., Newman J.D., Martin V.J., Keasling J.D. (2007). Microbial sensors for small molecules: Development of a mevalonate biosensor. Metab. Eng..

[19] Tang S.Y., Cirino P.C. (2011). Design and application of a mevalonate-responsive regulatory protein. Angew. Chem. Int. Ed. Engl..

[20] Liang W.F., Cui L.Y., Cui J.Y., Yu K.W., Yang S., Wang T.M., Guan C.G., Zhang C., Xing X.H. (2017). Biosensor-assisted transcriptional regulator engineering for Methylobacterium extorquens AM1 to improve mevalonate synthesis by increasing the acetyl-CoA supply. Metab. Eng..

[21] Chou H.H., Keasling J.D. (2013). Programming adaptive control to evolve increased metabolite production. Nat. Commun..

[22] Martin V.J., Pitera D.J., Withers S.T., Newman J.D., Keasling J.D. (2003). Engineering a mevalonate pathway in Escherichia coli for production of terpenoids. Nat. Biotechnol..

[23] Dahl R.H., Zhang F., Alonso-Gutierrez J., Baidoo E., Batth T.S., Redding-Johanson A.M., Petzold C.J., Mukhopadhyay A., Lee T.S., Adams P.D. (2013). Engineering dynamic pathway regulation using stress-response promoters. Nat. Biotechnol..

[24] Shen H.J., Cheng B.Y., Zhang Y.M., Tang L., Li Z., Bu Y.F., Li X.R., Tian G.Q., Liu J.Z. (2016). Dynamic control of the mevalonate pathway expression for improved zeaxanthin production in Escherichia coli and comparative proteome analysis. Metab. Eng..

[25] Henke N.A., Heider S.A.E., Hannibal S., Wendisch V.F., Peters-Wendisch P. (2017). Isoprenoid Pyrophosphate-Dependent Transcriptional Regulation of Carotenogenesis in Corynebacterium glutamicum. Front. Microbiol..

[26] Henke N.A., Austermeier S., Grothaus I.L., Gotker S., Persicke M., Peters-Wendisch P., Wendisch V.F. (2020). Corynebacterium glutamicum CrtR and Its Orthologs in Actinobacteria: Conserved Function and Application as Genetically Encoded Biosensor for Detection of Geranylgeranyl Pyrophosphate. Int. J. Mol. Sci..

[27] Amiri P., Shahpiri A., Asadollahi M.A., Momenbeik F., Partow S. (2016). Metabolic engineering of Saccharomyces cerevisiae for linalool production. Biotechnol. Lett..

[28] Yuan J., Ching C.B. (2015). Dynamic control of ERG9 expression for improved amorpha-4,11-diene production in Saccharomyces cerevisiae. Microb. Cell Fact.

[29] Callari R., Meier Y., Ravasio D., Heider H. (2018). Dynamic Control of ERG20 and ERG9 Expression for Improved Casbene Production in Saccharomyces cerevisiae. Front. Bioeng. Biotechnol..

[30] Ignea C., Raadam M.H., Motawia M.S., Makris A.M., Vickers C.E., Kampranis S.C. (2019). Orthogonal monoterpenoid biosynthesis in yeast constructed on an isomeric substrate. Nat. Commun..

[31] Kim S.K., Kim S.H., Subhadra B., Woo S.G., Rha E., Kim S.W., Kim H., Lee D.H., Lee S.G. (2018). A Genetically Encoded Biosensor for Monitoring Isoprene Production in Engineered Escherichia coli. ACS Synth. Biol..

[32] d’Oelsnitz S., Nguyen V., Alper H.S., Ellington A.D. (2022). Evolving a Generalist Biosensor for Bicyclic Monoterpenes. ACS Synth. Biol..

[33] Falcone Ferreyra M.L., Rius S.P., Casati P. (2012). Flavonoids: Biosynthesis, biological functions, and biotechnological applications. Front. Plant Sci..

[34] Panche A.N., Diwan A.D., Chandra S.R. (2016). Flavonoids: An overview. J. Nutr. Sci..

[35] Dias M.C., Pinto D., Silva A.M.S. (2021). Plant Flavonoids: Chemical Characteristics and Biological Activity. Molecules.

[36] Xu P., Marsafari M., Zha J., Koffas M. (2020). Microbial Coculture for Flavonoid Synthesis. Trends Biotechnol..

[37] Levisson M., Patinios C., Hein S., de Groot P.A., Daran J.M., Hall R.D., Martens S., Beekwilder J. (2018). Engineering de novo anthocyanin production in Saccharomyces cerevisiae. Microb. Cell Fact.

[38] Dudnik A., Gaspar P., Neves A.R., Forster J. (2018). Engineering of Microbial Cell Factories for the Production of Plant Polyphenols with Health-Beneficial Properties. Curr. Pharm. Des..

[39] Hwang E.I., Kaneko M., Ohnishi Y., Horinouchi S. (2003). Production of plant-specific flavanones by Escherichia coli containing an artificial gene cluster. Appl. Environ. Microbiol..

[40] Rogers J.K., Guzman C.D., Taylor N.D., Raman S., Anderson K., Church G.M. (2015). Synthetic biosensors for precise gene control and real-time monitoring of metabolites. Nucleic Acids Res..

[41] Siedler S., Khatri N.K., Zsohar A., Kjaerbolling I., Vogt M., Hammar P., Nielsen C.F., Marienhagen J., Sommer M.O.A., Joensson H.N. (2017). Development of a Bacterial Biosensor for Rapid Screening of Yeast p-Coumaric Acid Production. ACS Synth. Biol..

[42] Jiang T., Li C., Yan Y. (2021). Optimization of a p-Coumaric Acid Biosensor System for Versatile Dynamic Performance. ACS Synth. Biol..

[43] Li C., Zou Y., Jiang T., Zhang J., Yan Y. (2022). Harnessing plasmid replication mechanism to enable dynamic control of gene copy in bacteria. Metab. Eng..

[44] Marin A.M., Souza E.M., Pedrosa F.O., Souza L.M., Sassaki G.L., Baura V.A., Yates M.G., Wassem R., Monteiro R.A. (2013). Naringenin degradation by the endophytic diazotroph *Herbaspirillum seropedicae* SmR1. Microbiology.

[45] Wang R., Cress B.F., Yang Z., Hordines J.C., Zhao S., Jung G.Y., Wang Z., Koffas M.A.G. (2019). Design and Characterization of Biosensors for the Screening of Modular Assembled Naringenin Biosynthetic Library in Saccharomyces cerevisiae. ACS Synth. Biol..

[46] Zhou S., Yuan S.F., Nair P.H., Alper H.S., Deng Y., Zhou J. (2021). Development of a growth coupled and multi-layered dynamic regulation network balancing malonyl-CoA node to enhance (2S)-naringenin biosynthesis in Escherichia coli. Metab. Eng..

[47] Liu D., Sica M.S., Mao J., Chao L.F., Siewers V. (2022). A p-Coumaroyl-CoA Biosensor for Dynamic Regulation of Naringenin Biosynthesis in Saccharomyces cerevisiae. ACS Synth. Biol..

[48] Siedler S., Stahlhut S.G., Malla S., Maury J., Neves A.R. (2014). Novel biosensors based on flavonoid-responsive transcriptional regulators introduced into Escherichia coli. Metab. Eng..

[49] Espinosa-Urgel M., Serrano L., Ramos J.L., Fernandez-Escamilla A.M. (2015). Engineering Biological Approaches for Detection of Toxic Compounds: A New Microbial Biosensor Based on the Pseudomonas putida TtgR Repressor. Mol. Biotechnol..

[50] Teran W., Felipe A., Segura A., Rojas A., Ramos J.L., Gallegos M.T. (2003). Antibiotic-dependent induction of Pseudomonas putida DOT-T1E TtgABC efflux pump is mediated by the drug binding repressor TtgR. Antimicrob. Agents Chemother..

[51] Xiong D., Lu S., Wu J., Liang C., Wang W., Wang W., Jin J.M., Tang S.Y. (2017). Improving key enzyme activity in phenylpropanoid pathway with a designed biosensor. Metab. Eng..

[52] Sun H., Zhao H., Ang E.L. (2020). A New Biosensor for Stilbenes and a Cannabinoid Enabled by Genome Mining of a Transcriptional Regulator. ACS Synth. Biol..

[53] Daley S.K., Cordell G.A. (2021). Alkaloids in Contemporary Drug Discovery to Meet Global Disease Needs. Molecules.

[54] Ferreira M.U. (2022). Alkaloids in Future Drug Discovery. Molecules.

[55] Kopp T., Abdel-Tawab M., Mizaikoff B. (2020). Extracting and Analyzing Pyrrolizidine Alkaloids in Medicinal Plants: A Review. Toxins.

[56] Zhang J., Hansen L.G., Gudich O., Viehrig K., Lassen L.M.M., Schrubbers L., Adhikari K.B., Rubaszka P., Carrasquer-Alvarez E., Chen L. (2022). A microbial supply chain for production of the anti-cancer drug vinblastine. Nature.

[57] Zhao M., Qin Z., Abdullah A., Xiao Y. (2022). Construction of biocatalytic cascades for the synthesis of benzylisoquinoline alkaloids from p-coumaric acid derivatives and dopamine. Green Chem..

[58] Pyne M.E., Martin V.J.J. (2022). Microbial synthesis of natural, semisynthetic, and new-to-nature tetrahydroisoquinoline alkaloids. Curr. Opin. Green Sustain. Chem..

[59] DeLoache W.C., Russ Z.N., Narcross L., Gonzales A.M., Martin V.J.J., Dueber J.E. (2015). An enzyme-coupled biosensor enables (S)-reticuline production in yeast from glucose. Nat. Chem. Biol..

[60] Ricci V., Busby S.J., Piddock L.J. (2012). Regulation of RamA by RamR in Salmonella enterica serovar Typhimurium: Isolation of a RamR superrepressor. Antimicrob. Agents Chemother..

[61] Burstein S. (2015). Cannabidiol (CBD) and its analogs: A review of their effects on inflammation. Bioorg Med. Chem..

[62] Andre C.M., Hausman J.F., Guerriero G. (2016). Cannabis sativa: The Plant of the Thousand and One Molecules. Front. Plant Sci..

[63] Luo X., Reiter M.A., d’Espaux L., Wong J., Denby C.M., Lechner A., Zhang Y., Grzybowski A.T., Harth S., Lin W. (2019). Complete biosynthesis of cannabinoids and their unnatural analogues in yeast. Nature.

[64] Miettinen K., Leelahakorn N., Almeida A., Zhao Y., Hansen L.R., Nikolajsen I.E., Andersen J.B., Givskov M., Staerk D., Bak S. (2022). A GPCR-based yeast biosensor for biomedical, biotechnological, and point-of-use cannabinoid determination. Nat. Commun..

[65] Senchina D.S., Hallam J.E., Kohut M.L., Nguyen N.A., Perera M.A.d.N. (2014). Alkaloids and athlete immune function: Caffeine, theophylline, gingerol, ephedrine, and their congeners. Exerc. Immunol. Rev..

[66] Wachsmuth M., Findeiss S., Weissheimer N., Stadler P.F., Morl M. (2013). De novo design of a synthetic riboswitch that regulates transcription termination. Nucleic Acids Res..

[67] Crews C. (2015). Analysis of Ergot Alkaloids. Toxins.

[68] Wong G., Lim L.R., Tan Y.Q., Go M.K., Bell D.J., Freemont P.S., Yew W.S. (2022). Reconstituting the complete biosynthesis of D-lysergic acid in yeast. Nat. Commun..

[69] Gunsalus R.P., Yanofsky C. (1980). Nucleotide sequence and expression of Escherichia coli trpR, the structural gene for the trp aporepressor. Proc. Natl. Acad. Sci. USA.

[70] Gong X., Zhang R., Wang J., Yan Y. (2022). Engineering of a TrpR-Based Biosensor for Altered Dynamic Range and Ligand Preference. ACS Synth. Biol..

[71] van der Stel A.X., Gordon E.R., Sengupta A., Martinez A.K., Klepacki D., Perry T.N., Herrero Del Valle A., Vazquez-Laslop N., Sachs M.S., Cruz-Vera L.R. (2021). Structural basis for the tryptophan sensitivity of TnaC-mediated ribosome stalling. Nat. Commun..

[72] Bhatia S.K., Bhatia R.K., Yang Y.-H. (2016). Biosynthesis of polyesters and polyamide building blocks using microbial fermentation and biotransformation. Rev. Environ. Sci. Biotechnol..

[73] Chen X.F., Xia X.X., Lee S.Y., Qian Z.G. (2018). Engineering tunable biosensors for monitoring putrescine in Escherichia coli. Biotechnol. Bioeng..

[74] Childers M.C., Daggett V. (2017). Insights from molecular dynamics simulations for computational protein design. Mol. Syst. Des. Eng..

[75] Jumper J., Evans R., Pritzel A., Green T., Figurnov M., Ronneberger O., Tunyasuvunakool K., Bates R., Zidek A., Potapenko A. (2021). Highly accurate protein structure prediction with AlphaFold. Nature.

